# Achieving global surgical excellence: an evidence-based framework to guide surgical quality improvement programs in low and middle income countries

**DOI:** 10.3389/frhs.2023.1096144

**Published:** 2023-08-07

**Authors:** Jaymie Claire Henry, Lye-Yeng Wong, Ana M. Reyes, James Z. Jin, Mark K. Ferguson, Cheng Har Yip, Andrew Hill

**Affiliations:** ^1^ Department of Cardiothoracic Surgery, Baylor College of Medicine, Houston, TX, United States; ^2^ Department of Cardiothoracic Surgery, Stanford University, Palo Alto, CA, United States; ^3^ Department of Surgery, University of Miami/Jackson Health System, Miami, FL, United States; ^4^ Department of Surgery, The University of Auckland, Middlemore Hospital, Auckland, New Zealand; ^5^ Department of Cardiothoracic Surgery, University of Chicago, Chicago, IL, United States; ^6^ Department of Surgery, University of Malaya, Kuala Lumpur, Malaysia

**Keywords:** global surgery, patient safety, quality improvement, low- and middle-income countries, safe surgery

## Abstract

**Objectives:**

There is a lack of evidence-based guidelines for enhancing global surgical care delivery. We propose a set of recommendations to serve as a framework to guide surgical quality improvement and scale-up initiatives in low and middle income countries (LMICs).

**Methods:**

From January-December 2019, we reviewed the available literature and their application toward LMIC settings. The first initiative was the establishment of Best Practices Recommendations intended to summarize best-level evidence around quality improvement processes that have shown to decrease morbidity and mortality in LMICs. The GRADE level of evidence and strength of the recommendation were assigned in accordance with the *WHO handbook for guidelines development*. The second initiative was the scale-up of principles and practices by establishing international expert consensus on the optimal organization of surgical services in LMICs using a modified Delphi methodology.

**Results:**

Recommendations for three topic areas were established: reducing surgical site infections, improving quality of trauma systems, and interventions to reduce maternal and perinatal mortality. 27 studies were included in a quantitative synthesis and meta-analysis for interventions reducing surgical site infections, 27 studies for interventions improving the quality of trauma systems, and 14 studies for interventions reducing maternal and perinatal mortality. Using Delphi methodology, an international expert panel established consensus that district hospitals should place the highest priority on developing surgical services for low complexity, high volume conditions. At the national level, emergency and essential surgical care should be integrated within national Universal Health Coverage frameworks.

**Conclusions:**

This project fills a critical cap in the rapidly developing field of global surgery: gathering evidence-based, practical, and cost-effective solutions that will serve as a guide for the efficient planning and allocation of resources necessary to promote quality and safe essential surgical services in LMICs.

## 1. Background

Since 2015, increasing surgical capacity in low- and middle-income countries (LMICs) has received increased attention in the international community with the inception of several landmark initiatives including the Disease Control Priorities, third edition (DCP3), on Essential Surgery and the Lancet Commission on Global Surgery (1, 2). In response, many countries have undertaken the creation of national policies and initiatives to strengthen infrastructure for surgical systems (3, 4). Similarly, private organizations, non-governmental organization (NGOs), universities, academic societies, surgical colleges and other on-the-ground providers have worked to increase capacity for surgical, obstetric, trauma, and anesthesia care in LMICs (5–8). While strides have been made in the assessment of surgical capacity and the establishment of national priorities for enhancing surgical care delivery, there is a lack of evidence-based guidelines to guide the scale up of high quality and safe surgical, obstetric, trauma, and anesthesia care.

Quality improvement is essential to improving morbidity and mortality in surgical systems (9, 10). Kruk et al. published an impactful work assessing the quality of health systems in the Sustainable Goals Era and found that healthcare in LMICs is often inadequate, of variable quality, and exacerbated in vulnerable populations (11). Confidence in their healthcare system is a valuable judge of a nation's overall performance, and this paper reports that one in three people in LMICs indicated negative experiences with their respective healthcare systems with regard to accessibility, respect, and communication (11, 12). There is also significant variation in surgical outcomes, with adults up to three times, and children seven times more likely to die after emergency abdominal surgery in LMICs compared to high-income countries (HICs) (13). It is estimated that 23 million disability-adjusted life-years are lost each year due to in-hospital adverse events alone and that two-thirds of these occur in LMICs (10). One challenge to establishing international best practices is that the surgical landscape looks vastly different across settings based on existing health infrastructure and the number and distribution of health care providers (8, 10–12). Some countries are more mature in their surgical systems, with well-established trauma systems, outcomes surveillance, and effective referral systems (11, 12). Other countries may rely on a patchwork of organizations, each covering different domains in surgical service delivery (5, 7, 12). One of the keys to developing high quality healthcare systems is the ability to scale-up at a respectable rate without losing impact. White et al. performed a thorough systematic review and found that from 1960 to 2020, there were only 31 studies that reported scale-up interventions as quality improvement measures, with the implementation of the WHO surgical safety checklist as the most common intervention (14). Similarly in a systematic review by Brima et al, of the 49 articles that reported hospital-based quality improvement studies in Africa, use of the surgical safety checklist comprised 29% and reduction of surgical site infections comprised 25% (15). Other interventions well-known to high income settings such as antimicrobial stewardship programs and streamlined postoperative care protocols are lacking in LMICs (14, 15). As all countries move toward self-sufficient surgical systems, practical and adaptable guidelines are needed to inform quality improvement initiatives across unique national, regional, and local settings.

In December of 2018, the G4 Alliance and the International Society of Surgery (ISS/SIC) established the International Standards and Guidelines for Quality Safe Surgery and Anesthesia (ISG-QSSA) Working Group. The group consisted of clinicians, epidemiologists, Ministers of Health, and research methodologists. The group was tasked to gather and compile existing evidence-based guidelines and recommendations for quality improvement in LMICs. From January to December 2019, the ISG-QSSA held a series of meetings to review the available literature and their application toward LMIC settings. Ultimately, two research initiatives were established. The specific objectives of the Best Practice Recommendations initiative were to evaluate the literature on quality improvement interventions, processes, and structures which reduce mortality and morbidity in LMICs. A secondary objective was to evaluate the balance between harms and benefits of interventions, patient and provider preferences and concerns, and the feasibility of introducing the interventions into LMIC settings. Subsequently, the objective of the scale-up principles and practices initiative was to establish international expert consensus on a set of statements describing the optimal distribution and prioritization of surgical services in LMICs based on prior published evidence on the efficacy of decentralizing or regionalizing surgical services (16–19). The statements covered three major areas: (1) the optimal distribution of surgical services, (2) the optimal prioritization of surgical services, and (3) policies and practices for enhancing surgical scale-up. This paper describes the research initiatives and findings of the G4 Alliance and the International Society of Surgery (ISS/SIC) International Standards and Guidelines for Quality Safe Surgery and Anesthesia (ISG-QSSA) Working Group (20, 21). We propose a set of recommendations to serve as a framework to guide surgical quality improvement and scale-up initiatives in LMICs.

## 2. Methods

### 2.1. Best practice recommendations

A systematic review was undertaken by the ISG-QSSA Working Group using the PubMed, Embase, Cochrane, WHO regional databases, Google Scholar, and Grey literature databases to summarize the interventional data from LMICs that have shown to improve morbidity and mortality. Three systematic reviews were ultimately completed and recommendations were created based on the available evidence. To comply with current standards for evidence assessment in the formulation of policy recommendations, methodology adapted from the *WHO Handbook for Guidelines Development* was used (22). A Guideline Development Group, consisting of 8 experts in the field, were tasked to collate the most relevant and significant clinical questions and assign expert review to externally validate or reject the proposed questions. Review questions were developed within a framework population (restricted to LMICs), presence or absence of interventions under investigation, and mortality and morbidity outcomes. A consensus meeting was held in 2020 to confirm the findings of the systematic reviews and associated recommendation and assess the quality of evidence using GRADE methodology using independent reviewers (23–26). Of the eight criterion for rating the quality of evidence as described by the GRADE methodology, we focused on the three most dependable criteria as suggested by Malmivara et al. (24) We used risk of bias, inconsistency of findings, and publication bias to up or downgrade the quality of studies included (24, 25). Final recommendations were approved by the ISG-QSSA and formally ratified by the G4 Alliance Permanent Council on November 5th, 2021.

### 2.2. Scale-up principles and practices

33 international surgical experts convened in Suva, Fiji at a meeting hosted by the Fiji Ministry of Health in March 2020 where a roundtable discussion was held to refine modified Delphi statements around the topics of complexity, volume, and acuity of surgical care in LMICs. By providing input on their needs and the potential utility of the findings, 27 statements were collaboratively created which covered the global definitions of organization, distribution, and prioritization of surgical services in LMICs. Next, an open call was made for experienced surgeons and public health experts in the LMIC setting, and nominees by the G4 Alliance and Ministries of Health were sent a survey to confirm their credentials and experience. Using specific criteria such as: geographic scope, relevant expertise, work setting, and recognized impact, the final 53 participants were chosen representing 27 different LMICs. The experts were chosen for their recognized authority, clinical expertise in a range of surgical services, diverse geographical scope, and work in both public and private sectors. Half of the participants represented general surgery while the other half represented other surgical specialties. The participants were distributed across Africa, Asia, Europe, the Americas, and Oceania. A two-round Delphi process was used to establish consensus among this international panel of surgeon experts from LMICs. The first round involved independent ratings of the agreed upon statements without any interaction with the other participants. Participants chose the following options for each of the statements provided: strongly agree, agree, neutral, disagree, and strongly disagree. They were also instructed to make comments or edits to the proposed statements. The second round enabled participants to see de-identified comments from other involved members, but again, they provided ratings independently without engaging in discussion. The process was completed after two rounds because of the high rate of agreement between the 53 independent reviewers. Data was collected on an online platform, REDCap, which ensured anonymity during the two round process.

## 3. Results

### 3.1. Best practice recommendations

**Figure 1:**
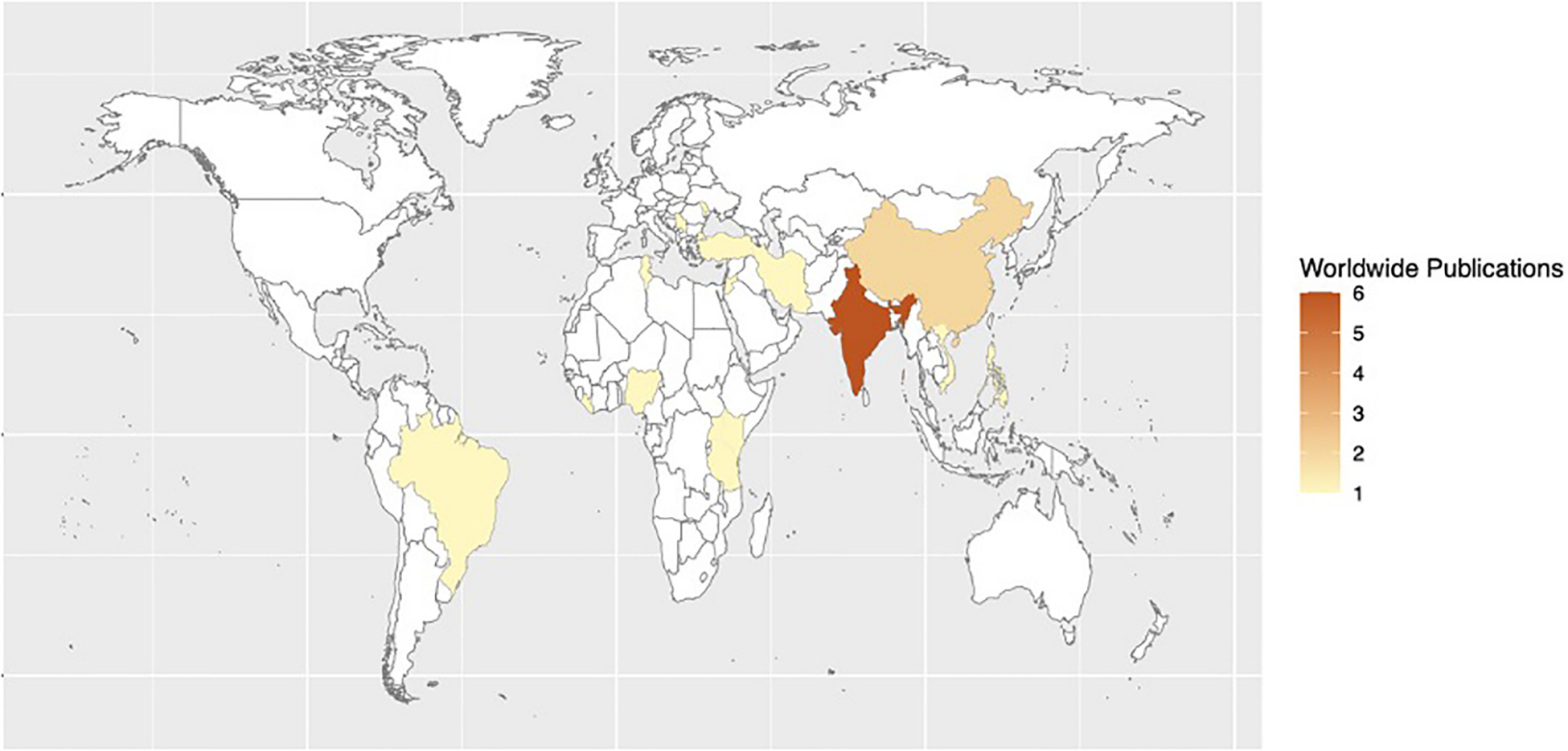
Heat map of studies assessing quality improvement processes, interventions, and structure for reducing morbidity and mortality from surgical site infections in LMICs.

**Figure 2:**
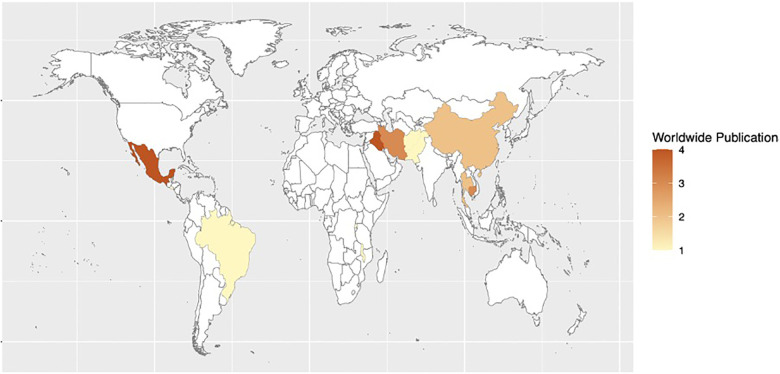
Heat map of studies assessing quality improvement processes, interventions, and structure of trauma systems in LMICs.

**Figure 3:**
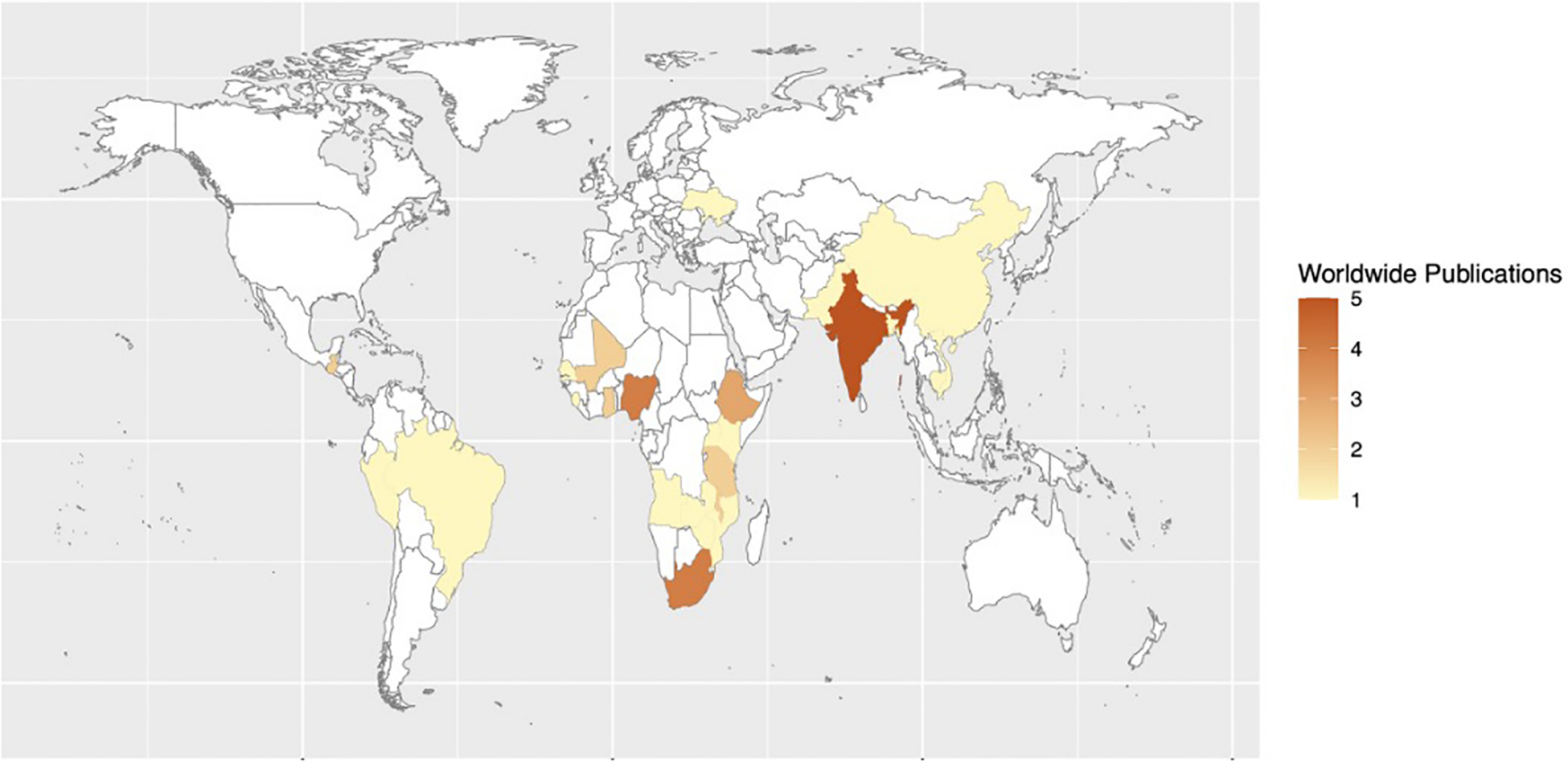
Heat map of studies assessing the impact of quality improvement processes, interventions, and structure on maternal and perinatal mortality in LMICs.

Recommendations covering three topic areas were established: (1) reducing surgical site infections, (2) improving quality of trauma systems, and (3) interventions to reduce maternal and perinatal mortality. For interventions reducing surgical site infections, 27 studies were included in a quantitative synthesis and meta-analysis. For interventions improving the quality of trauma systems, 27 studies were included. For interventions reducing maternal and perinatal mortality, 14 studies were included. The following heat maps demonstrate the countries in which research studies were conducted in each topic area (Figures 1–3).

Table 1 describes the final eleven Best Practice Recommendations. The GRADE level of evidence and strength of the recommendation are documented in accordance with the *WHO handbook for guidelines development*. A “strong” recommendation was made when it was clear that the net desirable consequences of the strategy outweighed those of no intervention. A “conditional” recommendation was made when it was less clear whether the net desirable consequences of the specified strategy outweighed those of no intervention.

**Table 1:** The G4 alliance best practice recommendations for quality improvement processes in LMICs.

Recommendation statement	GRADE certainty of evidence	Strength of recommendation
1. The G4 Alliance recommends the implementation of the WHO Surgical Safety Checklist to improve postoperative morbidity and mortality in low- and middle- income countries (LMICs)	High	Strong
2. The G4 Alliance recommends the establishment of a Hand Hygiene Programme in hospitals as a cost-effective measure to reduce the incidence of hospital acquired infections	Moderate	Strong
3. The G4 Alliance recommends the creation of an antimicrobial stewardship programme (ASP) alongside the development of antibiotic prophylaxis guidelines as a cost-saving strategy	Moderate	Strong
4. The G4 Alliance recommends the establishment of an appropriate prehospital trauma system to reduce morbidity and mortality in trauma patients in LMICs	Moderate	Strong
5. The G4 Alliance recommends the training of first responders to reduce morbidity and mortality in LMIC trauma patients.	Low	Strong
6. The G4 Alliance recommends the training of trauma providers to reduce morbidity and mortality in trauma patients in LMICs*.*	Moderate	Strong
7. The G4 Alliance recommends the institution of trauma audits as a quality improvement strategy to reduce trauma mortality and preventable trauma mortality in trauma patients in LMICs	Moderate	Strong
8. The G4 Alliance suggests the implementation of community-based programs in the training of traditional birth attendants (TBAs) to incorporate them into the health system in low-income countries where TBAs are acceptable and may be the sole provider for women in childbirth to reduce maternal and neonatal mortality rates	High	Conditional
9. The G4 Alliance recommends the implementation of maternal health quality improvement programs (such as maternal death reviews, combined with best practices implementation) to reduce hospital-based maternal mortality	High	Strong
10. The G4 Alliance recommends the upgrading of facilities and staff competencies to meet the standards for providing comprehensive emergency and newborn obstetric care (CEmONC) to increase the quality of care provided, decrease the number of unnecessary Caesarean sections, and decrease obstetric and newborn case fatality rates	Low	Strong
11. The G4 Alliance recommends the education and training (skills and drills training, simulation-based training, post-graduate training programs) of appropriate health workers (obstetricians, midwives, associate clinicians) in maternal and neonatal health in all levels of care to reduce maternal and child mortality	Moderate	Strong

### 3.2. Scale-up principles and practices

Recommendations covering three topic areas were established: (1) the optimal distribution of surgical services, (2) the optimal prioritization of surgical services, and (3) policies and practices for enhancing surgical scale-up. Table 2 recommends the organization and prioritization of surgical services based on complexity, volume, and acuity of procedures. Organization was categorized as regionalized vs. decentralized and prioritization was categorized as very high, high, or low.

**Table 2:** Recommendation matrix for surgical services based on complexity, volume, and acuity.

Complexity	Volume	Acuity	Example service	Organization	Priority for district center implementation	Comment
Low	High	Low	Preventive/screening Basic general surgery (e.g., hernia, common benign tumors) Basic ophthalmologic surgery (e.g., cataracts)	Decentralized	Very high	All communities need access to low complexity, high volume, low acuity surgical services. The low complexity of these services make them especially appropriate as the barriers to implementing them are lower.
Low	High	High	Basic trauma services Basic obstetric services Basic emergency surgery services (e.g., appendectomy)	Decentralized	Very high	Low complexity, high volume, high acuity services cannot be reasonably handled by referral. Regional centers have volume constraints and these problems are more efficient and cost-effective to handle at the district center, providing a chance for. improved outcomes. Basic services need to be available at the district center.
Low	Low	High	Basic emergency surgery services	Decentralized	High	Low complexity, low volume, high acuity services are best managed at the district center using basic services.
Low	Low	Low	Non-trauma orthopedic service	Decentralized	High	Low complexity, low volume, low acuity surgical services should be within the purview of a district center since it is a basic level of service.
High	High	Low	Common cancers (e.g., lung and breast cancer)	Regionalized	Low	High complexity, high volume, low acuity services are much needed in any community, but the high complexity and cost of implementing them becomes a lower priority. A proper screening and referral system at the community level should be implemented.
High	Low	Low	Complex oncologic and reconstructive services (e.g., pancreatic, liver cancer surgery)	Regionalized	Low	High complexity, low volume, low acuity services are served best by a national referral service.
High	Low	High	Complex emergency surgical services	Regionalized	Low	High complexity, low volume, high acuity services can be handled by a referral system.
High	High	High	Complex trauma services	Regionalized	Low	High complexity, high volume, high acuity services can adequately be handled by a system that can stabilize patients at a district center and transport them to a regional referral center.

#### 3.2.1. Recommendations for the optimal distribution of surgical services

The expert panel established consensus that low complexity surgical conditions should be decentralized, or managed by district centers close to communities. High complexity conditions should be regionalized or managed by specialized regional centers. In the case of trauma and emergency surgery, district centers should have the capacity to adequately stabilize patients and facilitate safe transfer of patients to regional centers for complex management.

#### 3.2.2. Recommendations for the optimal prioritization of surgical services

The expert panel established consensus that district hospitals should place the highest priority on developing surgical services for low complexity, high volume conditions. There was general agreement that managing these conditions at district centers would relieve tertiary centers of these demands. Respondents also recommended that district centers place high priority on developing services for low complexity, low volume conditions such as non-trauma orthopedic surgery.

Recommendations were also made for the role of district centers in triaging high complexity conditions. District centers should prioritize establishing systems for screening and referral of high complexity surgical cases to specialized centers. In the case of complex trauma, district centers should place priority on developing capacity to stabilize patients and facilitate transport to regional centers.

#### 3.2.3. Policies and practices for enhancing surgical scale-up

A set of principles for governments and organizations implementing surgical scale-up were developed. At the national level, emergency and essential surgical care should be integrated within national Universal Health Coverage (UHC) frameworks. National referral policies should be established to decrease delays in care, lower the cost of care, and improve outcomes. For surgical societies and university surgical programs in LMICs, in-country outreach is encouraged to reduce the backlog of neglected surgical diseases in underserved areas. Surgical societies or governments should establish registries and databases to better assess disease burden and specific facility performance, forming the backbone of performance assessment and monitoring.

### 3.3. Summary of recommendations

In summary, the ISG-QSSA proposes the following evidence-based recommendations to guide surgical quality improvement and scale-up in LMICs based on national, regional, district, and facility-level recommendations. On the national level, we propose integration of emergency and essential surgical care into national Universal Health Coverage (UHC) frameworks and the establishment of national referral policies that decrease delays in care, reduce costs, and improve outcomes. On the regional level, we propose the establishment of prehospital trauma systems, training of first responders, and training of trauma providers; regional center development of surgical services for high complexity cases; and the establishment of registries and databases to assess disease burden and facility performance, forming the backbone of quality improvement assessment and monitoring. On the district level, we propose district center development of surgical services for low complexity, high volume cases, procedures for stabilizing patients and facilitating safe and timely transfer in the case of complex trauma and emergency surgery, and the development and implementation of antibiotic prophylaxis guidelines. Lastly on the facility level, we propose the implementation of the WHO Surgical Safety Checklist, establishment of a hand hygiene program, creation of antimicrobial stewardship programs, upgrading of health care facilities to meet standards for providing comprehensive emergency and newborn obstetric care, implementation of maternal health quality improvement programs such as maternal death reviews, and institution of trauma audits.

## 4. Discussion

The recommendations put forth by this study are designed to provide a flexible framework that can be utilized by members of the global surgical community. They encompass surgery, anesthesia, trauma, and obstetrics recommendations, and utilize prioritization and distribution as a way to suggest both top down and bottom-up change. While all recommendations reflect evidence-based strategies to improve quality of surgical care in LMICs, individual recommendations pertain more specifically to unique tiers of a health care system. The designations of “national”, “region”, “district” and “facility-level” as mentioned above are flexible and can be defined more specifically according to the unique health system in which these recommendations will be applied. Additionally, individual recommendations may be relevant across multiple tiers and can be implemented in a parallel fashion, for example at both facility- and district-levels. Although the WHO highlighted the need to focus on scale-up over 15 years ago, there has been a lag between concept and reality (11, 12, 14). Implementation science education and experience may have been less common in decades past, but current research clearly indicates the need for these established frameworks to be at the center of any study design that aims to achieve sustainable implementation efforts (14, 27–29). With the matrix of recommendations reported in our study, we hope to add stepping stones that enables researchers, policymakers, and healthcare providers to create plans that synergize from each domain.

One of the strengths of this manuscript is the development of a set of building blocks that can help inform changes on both institutional and bureaucratic levels. The acknowledgement that there is no one size fits all technique to developed safe surgical systems is the first step to creating sustainable change. The WHO handbooks on diverse surgical topics have begun to set a foundation for strengthening surgical systems but there is more specificity needed to turn theory into action, which we hope to bridge with studies such as this (2, 10, 22, 29, 30). We aimed to contribute to existing literature by isolating publications from LMICs and following up with current knowledge, attitudes, and perceptions from LMIC experts who have the historical and current landscape of surgical care in their respective countries through a modified Delphi process. We envision the creation of a global implementation strategy for these recommendations that will complement the vast network of existing surgical communities. The G4 Alliance, as an organization, is working towards creating toolkits that will help individual hospitals, as well as national healthcare systems, enhance the already existing infrastructure to be able to practice and deliver healthcare at the highest of their capabilities (20). With the focus on surgery as an essential component of primary care, this solution-driven initiative will ultimately reduce the backlog of surgical neglected diseases by 2030 (27–30).

This study has several limitations including the fact the literature on quality improvement from LMICs is relatively sparse and does not include all the areas of perioperative care. Although the literature represented every region in the world, the geographic representations were skewed as resource limitation for research exists in certain areas of the world more than others. In addition, the members of the expert committee were predominantly from Africa and the situation may not be generalizable to distinct regions with unique baseline needs. Also, none of the panelists were trained specifically in obstetrics, while many do perform routine Cesarean sections. Nevertheless, the best practice recommendations covering maternal and perinatal care should be further vetted by LMIC obstetric experts. Most importantly, the majority of authors on this manuscript are from high-income countries. Although we received global participation during the multi-year process, the organization and drive for this manuscript ultimately stems from stakeholders in high-income countries, which highlights other issues in global surgery and collaborative research which we do not want to perpetuate. The Fiji Ministry of Health was actively involved in seeing the project through and we do aim to acknowledge the valuable time and effort involved for the coordination of the in-person portion of this study.

In this project, we started with a systematic review of the literature with the understanding that it may not be an accurate representation of healthcare systems in LMICs today. Research has shown that even with currently published data, we know very little about the actual patient experience in LMICs and the efficiency and competence of the overall healthcare system (11, 12, 14). Nevertheless, we do believe that having a starting point is essential to create targeted goals and inform new and higher quality research studies. We hope that this manuscript can serve as one tool in addressing the wide range of inequities and deficiencies across continents, not as a punitive measure, but as a call to action for both public and private sectors to work together in creating an achievable roadmap toward making a healthcare system that is for the people it serves. In conclusion, this project fills a critical cap in the rapidly developing field of global surgery: gathering evidence-based, practical, and cost-effective solutions that will serve as a guide for the efficient planning and allocation of resources necessary to promote quality and safe essential surgical services in LMICs.

## Data Availability

The raw data supporting the conclusions of this article will be made available by the authors, without undue reservation.
